# QuickStats

**Published:** 2015-02-27

**Authors:** FROM THE NATIONAL CENTER FOR HEALTH STATISTICS

**Figure:**
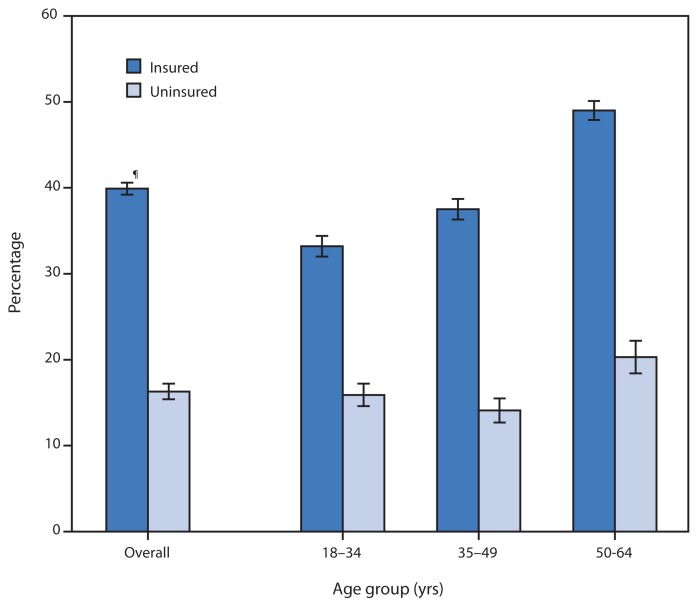
Percentage of Adults Aged 18–64 Years Who Have Seen or Talked with a Mental Health Professional in the Past 12 Months,* by Health Insurance Status^†^ and Age Group — National Health Interview Survey, United States, 2012–2013^§^ * Based on response to the question, “During the past 12 months, have you seen or talked to any of the following health care providers about your own health? A mental health professional such as a psychiatrist, psychologist, psychiatric nurse, or clinical social worker.” ^†^ Health insurance status is coverage at the time of interview. Persons were defined as uninsured if they did not have any private health insurance, Medicare, Medicaid, Children’s Health Insurance Program, state-sponsored or other government-sponsored health plan, or military plan. Persons also were defined as uninsured if they had only Indian Health Service coverage or had only a private plan that paid for one type of service. ^§^ Estimates are based on household interviews of a sample of the noninstitutionalized U.S. civilian population and are derived from the National Health Interview Survey sample adult component. ^¶^ 95% confidence interval.

During 2012–2013, the percentage of insured adults aged <65 years who reported seeing or talking with a mental health professional in the past 12 months was more than twice that of uninsured adults for all age groups. The percentage of adults generally increased with age for both insured and uninsured adults, with a larger increase occurring from persons aged 35–49 years to persons aged 50–64 years, for which the percentage increased from 37.5% to 49.0% for insured adults and from 14.1% to 20.3% for uninsured adults.

**Source:** National Health Interview Survey, 2012–2013. Available at http://www.cdc.gov/nchs/nhis.htm.

**Reported by:** Sandra L. Decker, PhD, sdecker@cdc.gov, 301-458-4748; Brandy J. Lipton, PhD.

