# Errata

**Published:** 2015-03-06

## Vol. 64, No. 7

In the QuickStats, “Percentage of Adults Aged 18–64 Years Who Have Seen or Talked with a Mental Health Professional* in the Past 12 Months, by Health Insurance Status^†^ and Age Group — National Health Interview Survey, United States, 2012–2013,^§^” data were incorrect. The corrected bar chart and text are below.

**Figure:**
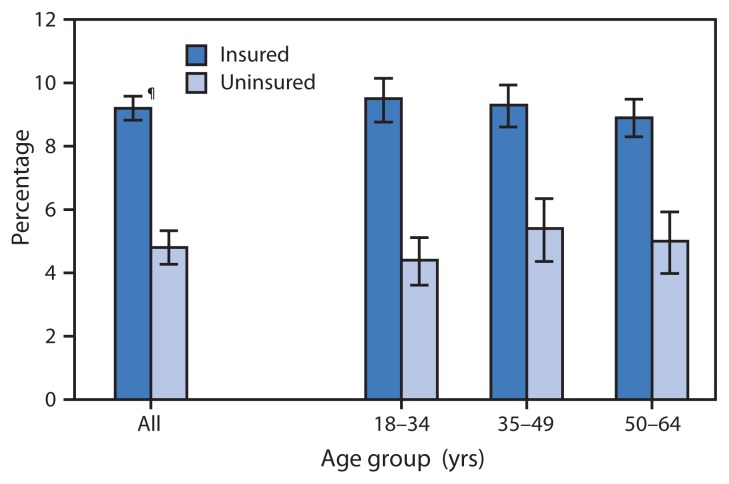
* Based on response to the question, “During the past 12 months, have you seen or talked to any of the following health care providers about your own health? A mental health professional such as a psychiatrist, psychologist, psychiatric nurse, or clinical social worker.” ^†^ Health insurance status is coverage at the time of interview. Persons were defined as uninsured if they did not have any private health insurance, Medicare, Medicaid, Children’s Health Insurance Program, state-sponsored or other government-sponsored health plan, or military plan. Persons also were defined as uninsured if they had only Indian Health Service coverage or had only a private plan that paid for one type of service. ^§^ Estimates are based on household interviews of a sample of the noninstitutionalized U.S. civilian population and are derived from the National Health Interview Survey sample adult component. ^¶^ 95% confidence interval.

During 2012–2013, the percentage of adults aged 18–64 years with health insurance who reported seeing or talking with a mental health professional in the past 12 months (9.2%) was approximately twice the percentage for uninsured adults (4.8%). The percentages of adults who reported seeing or talking with a mental health professional did not vary significantly by age group, and the difference between insured and uninsured adults was consistent across age groups.

**Source:** National Health Interview Survey, 2012–2013. Available at http://www.cdc.gov/nchs/nhis.htm.

**Reported by:** Sandra L. Decker, PhD, sdecker@cdc.gov, 301-458-4748; Brandy J. Lipton, PhD.

